# Effective Administration of Rituximab in Anti-MDA5 Antibody–Positive Dermatomyositis with Rapidly Progressive Interstitial Lung Disease and Refractory Cutaneous Involvement: A Case Report and Literature Review

**DOI:** 10.1155/2017/5386797

**Published:** 2017-10-31

**Authors:** Yuka Ogawa, Dai Kishida, Yasuhiro Shimojima, Koichi Hayashi, Yoshiki Sekijima

**Affiliations:** ^1^Department of Medicine (Neurology and Rheumatology), Shinshu University School of Medicine, 3-1-1 Asahi, Matsumoto 390-8621, Japan; ^2^Department of Dermatology, Shinshu University School of Medicine, 3-1-1 Asahi, Matsumoto 390-8621, Japan

## Abstract

We describe the case of a 48-year-old man with dermatomyositis (DM) who demonstrated rapidly progressive interstitial lung disease (RP-ILD) and refractory cutaneous involvement together with high levels of anti-melanoma differentiation-associated gene 5 antibody (anti-MDA5-Ab). Even after combination immunosuppressive therapy including a corticosteroid, cyclosporine A, and intravenous cyclophosphamide, his respiratory insufficiency and cutaneous involvement progressively worsened. However, the administration of rituximab (RTX) resulted in clinical remission as well as a visible reduction in anti-MDA5-Ab levels, suggesting that RTX could be a useful remedy in cases refractory to conventional immunosuppressive agents, especially those of RP-ILD related to anti-MDA5-Ab–positive DM.

## 1. Introduction

Dermatomyositis (DM) is an autoimmune inflammatory myopathy with characteristic cutaneous involvement such as Gottron's papules, a heliotrope rash, and/or an erythematous eruption around the neck and shoulders [1]. As a subtype of DM, clinically amyopathic DM (CADM), which is characterized by a typical skin lesion of DM with no or subclinical muscular manifestations, is substantially differentiated from classic DM [2]. In both CADM and classic DM, interstitial lung disease (ILD) has been recognized as the complication impacting on the prognosis; moreover, numerous studies have recently described that the development of rapidly progressive ILD (RP-ILD) is implicated in the positivity of anti-melanoma differentiation-associated gene 5 antibody (anti-MDA5-Ab), which is more frequently detected in patients with CADM than in those with classic DM [3–6].

Although combination immunosuppressive therapy consisting of a corticosteroid, calcineurin inhibitor, and intravenous cyclophosphamide (IVCY) is sometimes selected to prevent patients with DM-related RP-ILD from developing fatal disease, such an intensive therapeutic strategy is not entirely sufficient to ensure a favorable prognosis [7–9]. Rituximab (RTX), a chimeric monoclonal antibody for depleting B cells showing CD20 protein, was recently demonstrated to be effective for intractable muscular and/or cutaneous involvement in polymyositis or DM [10, 11]. It was also suggested that RTX could be useful for severe ILD in antisynthetase syndrome [12, 13]; meanwhile, there are only a few case reports in which RTX was used in anti-MDA5-Ab–positive DM with ILD [14–18].

Here we describe a case in which RTX ameliorated RP-ILD as well as refractory cutaneous involvement in a patient with anti-MDA5-Ab–positive DM despite the resistance to the conventional immunosuppressive therapy. We also review the literature for studies of RTX in anti-MDA5-Ab–positive DM with ILD.

## 2. Case Presentation

**Figure 1:**
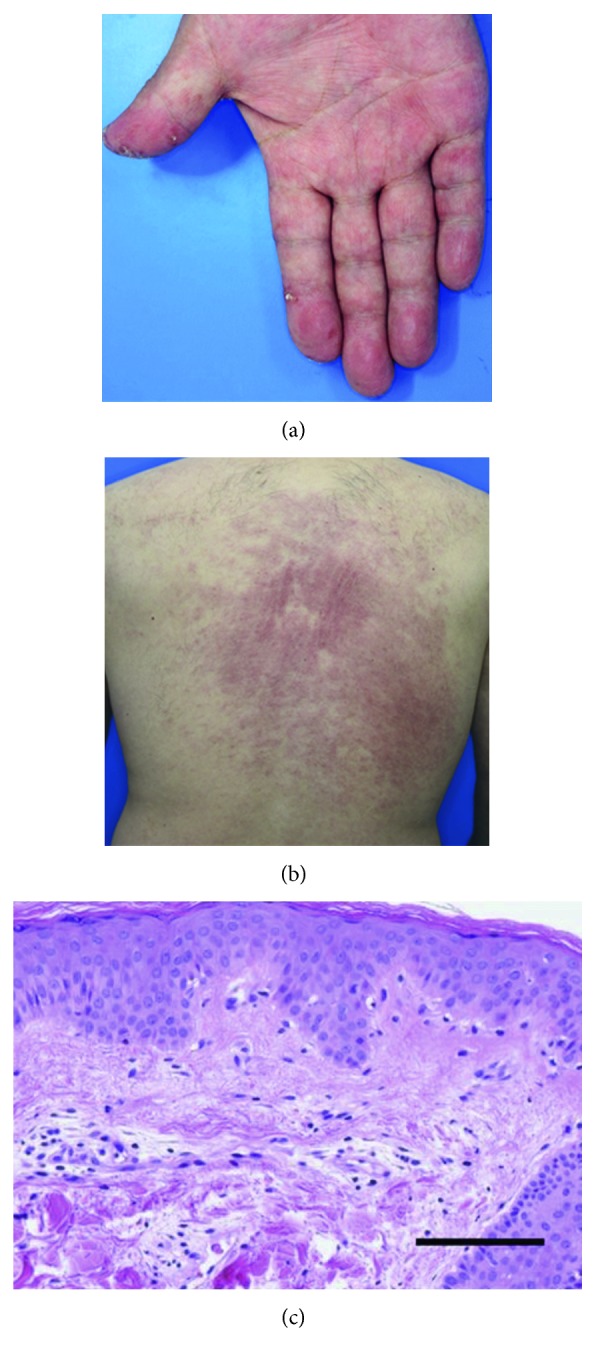
Skin lesions including palmar papules (a) and erythema on the back (b) before the initiation of treatment. A skin biopsy from the back indicates liquefactive degeneration with perivascular inflammation between the epidermis and dermis (c) (hematoxylin and eosin staining; scale bar = 100 µm).

**Figure 2:**
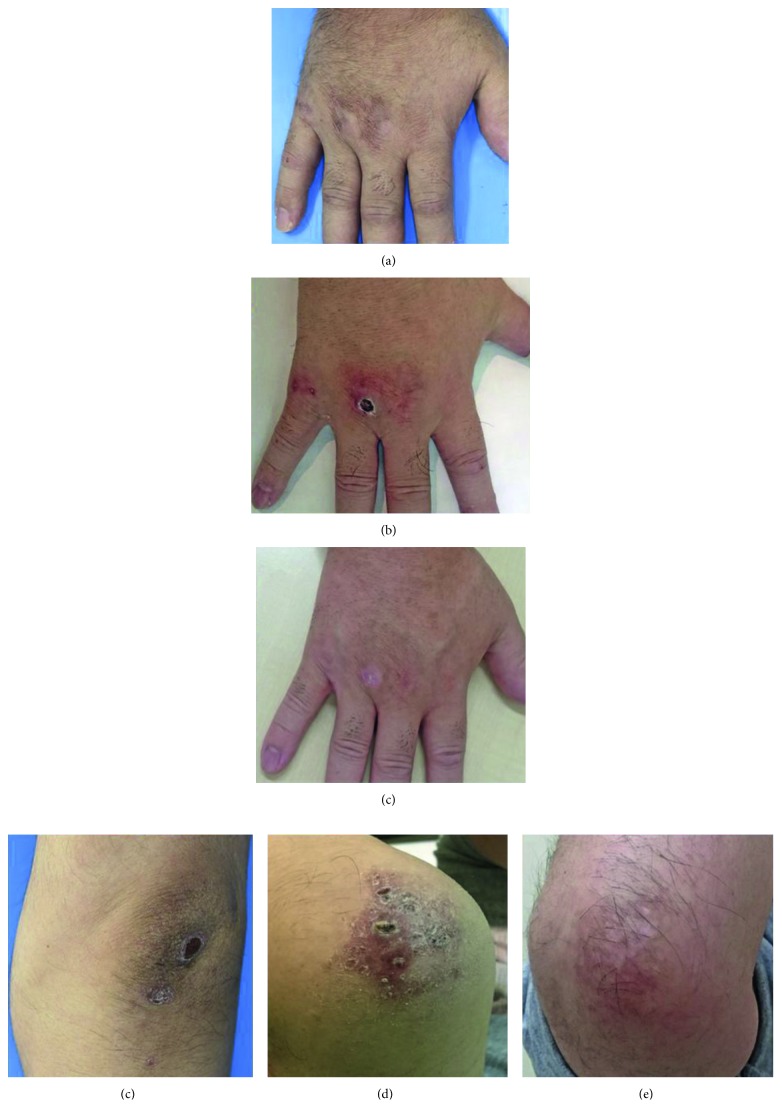
Sequential findings of cutaneous lesions on the dorsum of the hand and elbow before the initiation of treatment (a, d), before the addition of rituximab (RTX) (b, e), and after RTX administration (c, f).

**Figure 3:**
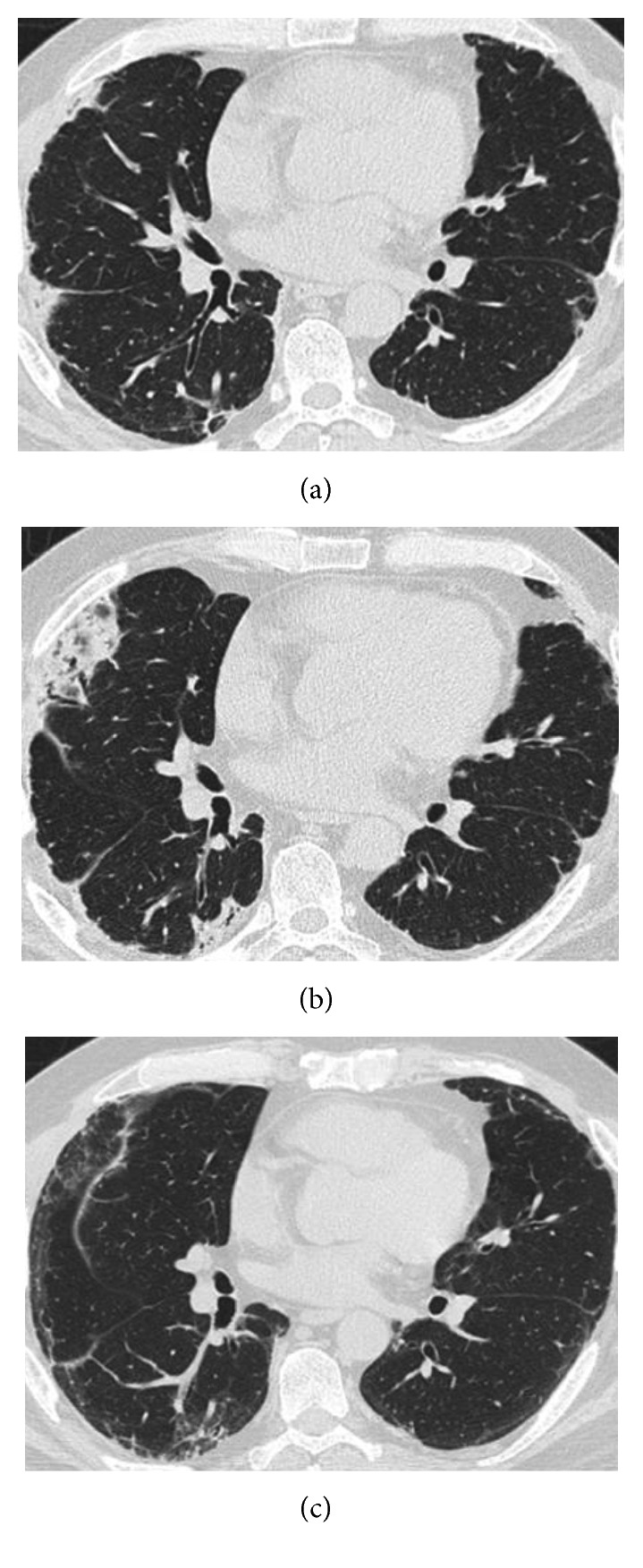
Chest computed tomography findings at admission (a), before the addition of rituximab (RTX) (b), and after RTX administration (c).

A 48-year-old man with a 1-month history of fatigue, appetite loss, and fever was admitted to our hospital. He reported experiencing arthralgia and a dry cough as well as mild exertional dyspnea prior to admission. A physical examination demonstrated a body temperature of 37.7°C, mild muscular weakness of the proximal lower limbs, edematous hands, and cutaneous manifestations including a heliotrope rash, Gottron's papules, mechanic's hands, palmar papules, and an erythematous rash on his face and back; in particular, ulcerative and erosive erythema was visible on his elbows (Figures 1 and 2). The pathological finding of the biopsied skin from his back demonstrated perivascular infiltration of inflammatory cells with liquefactive degeneration through the epidermis to the dermis (Figure 1(c)). Laboratory examinations revealed elevated serum levels of creatine kinase (CK) (278 U/L; normal, 43–230), C-reactive protein (0.59 mg/dL; normal, <0.10), ferritin (781 ng/mL; normal, 25–280), Krebs von den Lungen-6 (602 U/mL; normal, 105–435), and lactate dehydrogenase (453 U/L; normal, 120–230). Anti-aminoacyl-tRNA synthetase (anti-ARS) antibodies were not detected; meanwhile, a high titer of anti-MDA5-Ab was seen (>150 indexes; normal, <32). The detection of anti-MDA5-Ab was performed by enzyme-linked immunosorbent assay. Tests for anti-nuclear antibody, rheumatoid factor, anti-citrullinated protein antibody, and anti-neutrophil cytoplasmic antibodies specific for either myeloperoxidase or proteinase-3 were negative. Arterial blood gas analysis revealed a PaO_2_ of 66.4 mmHg and PaCO_2_ of 32.9 mmHg on room air. Chest computed tomography (CT) revealed reticular shadows and ground-glass opacity on the middle to inferior fields of the bilateral lung (Figure 3(a)).

**Figure 4:**
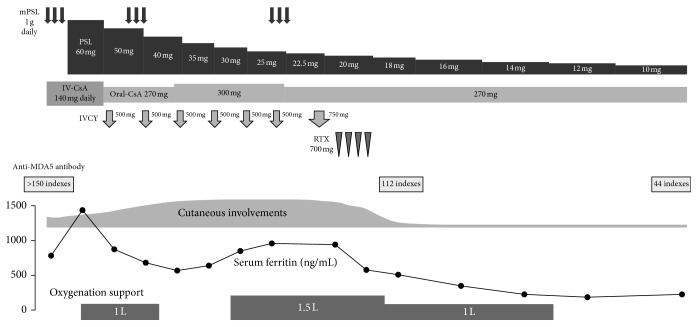
Clinical course of this patient. mPSL, methylprednisolone; PSL, prednisolone; IV-CsA, continuous intravenous infusion of cyclosporine A; oral-CsA, oral administration of cyclosporine A; IVCY, intravenous infusion of cyclophosphamide; RTX, rituximab.

Since the patient was diagnosed with DM-related ILD and a low PaO_2_, methylprednisolone (mPSL) (1 g daily for 3 consecutive days) was immediately administered together with a continuous intravenous infusion of cyclosporine A (IV-CsA) according to the previously described therapeutic protocol [9] (Figure 4). Subsequently, prednisolone (PSL) at the dose of 60 mg (1 mg/kg) daily and cyclosporine A, the blood trough concentration of which was adjusted to 150–200 ng/mL, were orally administered after mPSL and IV-CsA therapy, resulting in improved fatigue, appetite loss, fever, edema of the hands, eruptions on the face and back, and arthralgia. Muscle weakness also recovered, and serum CK level normalized. Meanwhile, respiratory insufficiency persisted, and oxygenation support was ultimately required. Therefore, IVCY was additionally administered at the dosage of 500 mg every 2 weeks for 6 cycles. However, his respiratory status was gradually worsening even after additional administration of 750 mg of IVCY. Moreover, further exacerbation of the chest CT finding was obviously demonstrated (Figure 3(b)). In addition, ulcerative and erosive eruptions on his hands and elbows also deteriorated (Figures 2(b) and 2(e)). On the 125th day since the initiation of the therapy, RTX was administered at the dosage of 700 mg (375 mg/m^2^) weekly for total 4 weeks. Consequently, cutaneous involvements on his hands and elbows were remarkably improved (Figures 2(c) and 2(f)), and recovery from respiratory insufficiency and amelioration of the radiographic finding were also achieved (Figure 3(c)). Furthermore, serum levels of ferritin and anti-MDA5-Ab decreased to 186 ng/mL and 44 indexes, respectively (Figure 4).

## 3. Discussion

This patient was found to be in the severe stage of ILD associated with DM because of having some prognostic factors including not only hypoxemia with anti-MDA5-Ab positivity but also refractory ulcerative eruptions as well as palmer papules. In fact, it has been previously described that existing cutaneous ulceration and/or palmer papules are associated with acute progression of ILD in DM [6, 7]; furthermore, the positive correlation between severity of ulcerative cutaneous involvement and anti-MDA5-Ab has been demonstrated [6, 19, 20]. In addition, anti-MDA5-Ab positivity is associated with fever and arthritis [19, 20], which were also revealed prior to initiating treatment in the present case. Therefore, immediate and intensive immunosuppressive therapy was required in this patient in order to avoid a fatal situation. It has been recognized that the concomitant use of CsA or tacrolimus with PSL is indispensable to prevent the progression of ILD in DM [21–23]; furthermore, additional administration of IVCY should be required in the acute exacerbation of the disease [7, 9, 23]. However, initial combination therapy, including PSL, CsA, and IVCY, was eventually insufficient for healing both cutaneous involvement and ILD. On the other hand, RTX therapy obviously contributed to achieving a favorable outcome.

MDA5 plays a crucial role in inducing an innate immune response against viral infection and is also recognized as the specific autoantigen in DM, suggesting that upregulation of MDA5 in the innate immune system subsequently promotes anti-MDA5-Ab production [24]. The production of specific autoantibodies in autoimmune diseases is attributed to autoreactive B cells; furthermore, RTX is found to succeed in depleting the pathogenic autoreactive B cells which secrete a specific autoantibody in several autoimmune diseases [25]. Even though autoreactive B cells also play the roles in activating effector T cells or producing proinflammatory cytokines, RTX may be indirectly implicated in preventing these immunological factors from attacking the target organ [25–27]. Interestingly, the recent overviewed study demonstrated that favorable therapeutic efficacy of RTX could be obtained in majority of patients with inflammatory myositis who had disease-specific autoantibodies [28]. It was described that high titer of anti-MDA5-Ab is associated with the severity of disease [29, 30]. On the other hand, subsequent reduction of anti-MDA5-Ab after starting the treatment means obviously predicting a successful outcome [20]. Although this patient initially had a level of anti-MDA5-Ab over the upper measurable limit, RTX administration reduced the levels of anti-MDA5-Ab and serum ferritin, which is also known as the prognostic factor in DM-related RP-ILD [31] (Figure 4), demonstrating that RTX therapy could correspondingly contribute to the recovery from severe clinical situations and suppress the production of anti-MDA5-Ab. Given the relationship between the therapeutic mechanism of RTX and autoantibodies including anti-MDA5-Ab, RTX therapy may be a reasonable option for achieving a favorable outcome, especially in RP-ILD related to anti-MDA5-Ab–positive DM.

To our knowledge, six cases treated with RTX in anti-MDA5-Ab–positive DM with ILD have been reported in the English literature to date [14–18] (Table 1). Their clinical characteristics were CADM or minimal muscular manifestations, the latter of which is consistent with the present case. One patient was also given RTX to treat the refractory cutaneous involvement even after ILD remission induced by the prior immunosuppressive therapy, whereas others required RTX because the preceding use of other immunosuppressive agents was ineffective in suppressing the exacerbation of ILD with or without cutaneous involvement. However, only half of them recovered from the RP-ILD, suggesting that it may be necessary to initiate effective therapy within the reversible state of disease even if RTX is a potential remedy in cases that are resistant to the prevalent immunosuppressive therapies.

Anti-MDA5-Ab–positive DM usually emerges with refractory cutaneous involvement and/or RP-ILD as a life-threatening complication. The present case demonstrated that RTX could be a useful therapy for achieving a favorable outcome. On the other hand, further clinical experiences must be accumulated to establish the therapeutic strategy using RTX for this disease.

## Figures and Tables

**Table 1:** Summarized clinical profiles in patients of anti-MDA5-antibody–positive DM with ILD who were treated with rituximab.

Ref. no.	Sex/age	Preceding manifestations				Previous treatment	Admission/after RTX [maximum^1^]			RTX targeting lesion	Duration prior to RTX^2^	RTX dosage (cycles)	Therapy during or after RTX	Outcome
		Respiratory (ILD)	Muscular (CK levels)	Cutaneous	Others		Ferritin (ng/mL)	KL-6 (U/mL)						
[14]	F/68	Cough, unusual dyspnea	Generalized weakness (normal)	Raynaud, erythema on the face, back, limbs, and hands	Fever, arthralgia, appetite loss	mPSL, PSL, IVIg, IVCY, MMF, CsA, Tac (topical), HCQ	805/n.d.	n.d./n.d.	Cutaneous lesion		About 2 years	1000 mg, per 15 days (×2)	n.d.	Improved^3^
[15]	F/58	n.d.	Weakness on the deltoids (normal)	Heliotrope rash, Gottron's papules	n.d.	PSL, Tac, IVCY	891.7/n.d.	613/2756 [4185]	ILD		About 3 months	500 mg, 375 mg/m^2^/week (×4)	mPSL, PSL, IVCY, IVIg, PMX	Improved
[16]	F/55	Rapidly progressive shortness of breath	None (normal)	Raynaud, heliotrope rash, Gottron's papules, rash on the hands	Weight loss	mPSL	n.d./n.d.	n.d./n.d.	ILD		n.d.	n.d.	CPA, PE	Died
[17]	F/71	Rapid deterioration of respiratory status, hypoxia	None (211 U/L)	Heliotrope rash, Gottron's papules, ulcer on the buttocks, papules on the fingers and elbows	Appetite loss, fatigue	mPSL, PSL, IVCY, IVIg, PMX	1782.8/253.1 [3149.8]	666/^4^	ILD cutaneous lesion		102 days	525 mg, 350 mg/m^2^/week (×4)	PSL, Tac	Improved
[18]	F/71	Dry cough, continuous deterioration of respiratory status	None (n.d.)	Purpura on the elbows, erythema on the anterior chest	Fever	mPSL, PSL, Tac, CsA, IVCY	507/1740 [1740]	991/n.d.	ILD		38 days	600 mg, 375 mg/m^2^/week (×2)	mPSL, PSL, CsA, MMF, Tac	Died
[18]	F/69	Exertional dyspnea, respiratory distress with hypoxia	None (225 U/L)	Gottron's papules, rash on the extremities, hyperkeratosis on the palmer side of fingers	Arthralgia	mPSL, PSL, CsA	219/1930	922/1520	ILD		33 days	500 mg, 375 mg/m^2^/week (×2)	mPSL, PSL, CsA, IVCY, tocilizumab, CHD	Died
This case	M/48	Exertional dyspnea with hypoxia, dry cough	Mild weakness on the lower limbs (278 U/L)	Heliotrope rash, Gottron's papules, mechanic's hands, palmar papules, erythema on the face and back, ulcer/erosion on the elbows	Fatigue, fever, appetite loss, arthralgia	mPSL, PSL, CsA, IVCY	781/186 [1437]	602/638 [1674]	ILD cutaneous lesion		125 days	700 mg, 375 mg/m^2^/week (×4)	PSL, CsA	Improved

DM, dermatomyositis; ILD, interstitial lung disease; Ref., reference; CK, creatine kinase; n.d., not described; Raynaud, Raynaud phenomenon; RTX, rituximab; mPSL, methylprednisolone; PSL, prednisolone; IVIg, intravenous immunoglobulin; IVCY, intravenous cyclophosphamide; MMF, mycophenolate mofetil; CsA, cyclosporine A; Tac, tacrolimus; HCQ, hydroxychloroquine; CPA, cyclophosphamide; PMX, polymyxin B hemoperfusion treatment; PE, plasma exchange; CHD, continuous hemodiafiltration. ^1^Maximum value if it was described in the report. ^2^Duration prior to administering RTX since initiating hospitalization. ^3^Remission of painful erythematous papules on the hands was obtained [14]. ^4^Decrease of KL-6 levels after RTX administration was shown in the figure of the described report [17].
